# Changes to Pork Bacterial Counts and Composition After Dielectric Barrier Discharge Plasma Treatment and Storage in Modified-Atmosphere Packaging

**DOI:** 10.3390/foods13244162

**Published:** 2024-12-22

**Authors:** Yi Zhou, Huixin Zuo, Zhaoqi Dai, Zonglin Guo, Benjamin W. B. Holman, Yanqin Ding, Jingying Shi, Xiaoxiao Ding, Mingming Huang, Yanwei Mao

**Affiliations:** 1 Key Laboratory of Food Processing Technology and Quality Control in Shandong Province, College of Food Science and Engineering, Shandong Agricultural University, Tai’an 271018, China; ytuzy2023@163.com (Y.Z.); hxzuo@sdau.edu.cn (H.Z.); jyshi80@163.com (J.S.); 19806101565@163.com (X.D.); maoyanwei@163.com (Y.M.); 2 College of Biotechnology, Jiangsu University of Science and Technology, Zhenjiang 212100, China; dai0003199@jsafc.edu.cn; 3 College of Food Science, South China Agricultural University, Guangzhou 510642, China; gzl072810@scau.edu.cn; 4 Wagga Wagga Agricultural Institute, NSW Department of Primary Industries, Wagga Wagga, NSW 2650, Australia; benjamin.holman@dpi.nsw.gov.au; 5 College of Biotechnology, Shandong Agricultural University, Tai’an 271018, China; dyq@sdau.edu.cn

**Keywords:** cold plasma, pork, sublethal injury, refrigerated storage, indigenous microbiota populations, spoilage bacteria interaction, modified culture methods

## Abstract

The aim of this study was to compare the succession of natural microbiota in pork held under refrigerated storage for up to 10 days after dielectric barrier discharge (DBD) plasma treatment. Two methods were used to assess the impact of DBD on microorganisms. Firstly, traditional selective media (SM) were employed to detect the bactericidal effects of DBD on *Pseudomonas* spp., *Enterobacteriaceae*, *Lactic acid bacteria* (LAB), and *Brochothrix thermosphacta*. Secondly, the thin agar layer (TAL) method was used to further evaluate the bactericidal effects of DBD. In addition, the Baranyi and Roberts model was applied to explore the kinetic parameters of *Pseudomonas* spp., *Enterobacteriaceae*, LAB, and *B. thermosphacta* during storage. Finally, the modified Lotka–Volterra model was used to describe the interactions between each microorganism. The study found that when using traditional selective media (SM), 85 kV DBD had a significant bactericidal effect on *Pseudomonas* spp., *Enterobacteriaceae*, LAB, and *Brochothrix thermosphacta*. However, when using the thin agar layer (TAL) method, the results suggested that DBD had no significant bactericidal effect, suggesting that DBD caused sublethal damage to the natural microorganisms on pork. Analysis with the Baranyi and Roberts model showed that DBD treatment significantly extended the lag phase of these four types of microorganisms and significantly reduced the μ_max_ of all microorganisms except LAB. The analysis results of the modified Lotka–Volterra model showed that LAB had a greater impact on *Pseudomonas* spp., *Enterobacteriaceae*, and *B. thermosphacta* (a_21_ > a_12_). In conclusion, DBD treatment was shown to have a significant sublethal bactericidal effect that impacted both the count and composition of natural microorganisms found on pork.

## 1. Introduction

Demand for high-quality and safe meat has grown over the past 50 years, and in response, the production of meat has more than tripled to ~340 million tons per year. The same trend is apparent for pork, with consumers preferencing its nutritional and organoleptic properties—although these same properties, its high level of water activity, and moderate pH make pork highly susceptible to microbial contamination and proliferation [1]. Microorganisms can cause the degradation of proteins, carbohydrates, and other physiochemical components of pork and thereby result in the development of malodors, structure/texture degradation, and discoloration [2]. Microorganisms on pork also represent a potential risk to the consumer, as many microorganisms are associated with foodborne disease and health complications, and infer a socioeconomic cost when ingested [3]. For these reasons, consumers are increasingly conscious about food safety and its processing methods being free from chemical sanitizers and artificial additives. Non-thermal sterilization technologies should therefore be considered novel methods for controlling microorganisms on pork and pork products [4].

Cold plasma has emerged as a sanitizing technology with considerable potential in its application to the preservation of foods [5,6]. Cold plasma is an ionized gas that is highly energetic and composed of ions, electrons, neutral atoms, and free radicals. Among the various constituents in cold plasma, the reactive species (reactive oxygen species and reactive nitrogen species) are regarded as the major agents for plasma-induced bactericidal effects [7]. It is generally believed that these reactive species, independently or synergistically, cause the destruction of the cell membrane, the dysfunction of proteins, damage to DNA, the peroxidation of lipid barriers, and disturbances to cellular homeostasis—actions that can effectively sterilize a food product [8,9,10]. Cold plasma devices have low energy inputs, high sterilization efficiency, and non-toxic residue characteristics, allow for the good retention of produce quality attributes, and are operated at room temperature [10,11,12,13]. These characteristics promote its application in the processing of meat products. Jayasena et al. [14] reported that exposure to flexible thin-layer cold plasma allowed for the effective inactivation of pathogens inoculated on beef jerky. Likewise, Moutiq, Misra, Mendonca and Keener [15] found that exposure to atmospheric cold plasma could decontaminate chicken breasts and deliver a 2 log CFU/g reduction in microorganism levels. Although cold plasma has been verified to efficiently inactivate microorganisms, its sublethal effect on microorganisms should also be considered.

Sublethal injury refers to microorganisms that have been exposed to chemical or physical stress and are consequentially damaged but not killed, the damage being structural (i.e., cell morphology, membrane integrity), metabolic (i.e., cellular homeostasis, membrane potential), or a combination of both [16,17,18]. Conventional analytical methods used in food-microbial diagnostics contain a variety of selective compounds that may be harmful to injured cells and contribute to type II error. On the other hand, under optimal environmental conditions, the rejuvenation of injured bacteria can increase the risk of underestimating the microbiological quality and safety of products during storage and distribution [19]. Initially, the inactivation of microorganisms triggered by membrane damage, due to non-thermal treatment, were deemed ‘all-or-nothing’, which means that cells without sublethal injury are generated [8]. However, research has demonstrated that cold plasma treatment could induce reversible cellular damage to microorganisms. Laroussi, Richardson and Dobbs [20], for example, reported that normal cell functioning is altered upon exposure to plasma and may return after a period of recovery, demonstrating reversible damage in cells exposed to plasma. Critzer, Kelly-Wintenberg, South and Golden [21] also found that the recovery of plasma-treated *Salmonella* on a selective medium (SM, xylose lysine tergitol-4 agar) was poorer than its recovery on the non-selective medium (TSAN). Microorganisms with sublethal injury often lose the ability to form visible colonies on a selective medium (SM), but they may recover and subsequently reproduce on a non-selective medium. This is important as previous research shows that cold plasma treatment can induce the sublethal injury of microorganisms [16,22,23,24]. Most of these studies have investigated microorganisms in vitro or via an inoculated food matrix. Furthermore, the sublethal effect of cold plasma on indigenous microorganisms of food remains to be understood—specially, there is limited information on the dynamic changes in the microbial communities of cold-plasma-treated pork held under refrigerated storage conditions and in modified-atmosphere packaging [25].

This study aimed to compare the effect of dielectric barrier discharge (DBD) plasma treatment on the indigenous microorganisms of pork during its refrigerated storage. The sublethal effects of plasma treatment on microorganisms were examined using the SM and thin agar layer (TAL) culture methods. The kinetics parameters of the indigenous microorganisms were determined using a modified Baranyi and Roberts model, and the antagonistic activities among different microorganisms were explored using a modified Lotka–Volterra model.

## 2. Materials and Methods

### 2.1. Meat Sample Preparation

**Figure 1:**
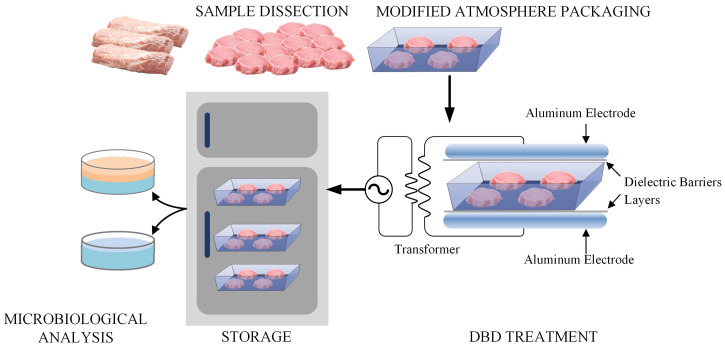
A schematic diagram of the experimental design used for the cold plasma treatment of pork samples held under refrigerated storage for up to 10 days, post-treatment. After the meat is trimmed, it undergoes modified-atmosphere packaging (MAP) with a composition of (80% O_2_ and 20% N_2_). Following the MAP, the products are treated with plasma at different voltages. The treated samples are then stored and sampled at various points for microbial analysis.

Figure 1 provides a schematic diagram of the experimental factors applied to each of the three replicates (chronological repeats). Within each replicate, three *M. longissimus thoracis et lumborum* (LTL, average weight: 6.5 kg) were purchased from a commercial retail outlet (Suguo Supermarket Co., Ltd., Nanjing, China). These were refrigerated overnight at 4 °C before trimming under aseptic conditions to remove all the external fat. From each LTL (216 per replicate), a total of 72 sample cores (weight: ~50 g, thickness: 2.0 cm, diameter: 5.5 cm) were prepared, under aseptic conditions and using a hollow stainless-steel cylinder. These 72 sample cores were randomly allocated into groups of 4, which were packaged together (18 trays per LTL) in a polypropylene tray (HS-6; Shanghai Chuo Kagaku Co., Ltd., Shanghai, China). The trays were flushed with an 80% O_2_ and 20% N_2_ gas mixture and heat sealed with polyamide/polyethylene barrier film (oxygen transmission rate of 3 cm^3^/m^2^/24 h) using a modified-atmosphere packaging machine (Senrui H360, Suzhou Senrui Fresh Keeping Equipment Co., Ltd., Suzhou, China). The in-pack gas compositions were verified with a gas analyzer (Check Point-Handheld Gas Analyzer, Ringsted, Denmark); the headspace compositions were found to be 80.1 ± 2.3% O_2_, and 19.9 ± 2.4% N_2_. All the sealed trays were placed in a refrigerated room for 2 h, utilizing the RH/Temp data logger R-4HC to ensure that in-pack relative humidity could stabilize at >80% (Elitech Inc., Fujian, China).

Within the replicates (54 trays per replicate) and balanced by LTL (18 trays per LTL), 3 trays were randomly allocated to each combination of 3 DBD treatments (0 kV (control) 60 kV, or 85 kV DBD) and 6 storage periods (0, 2, 4, 6, 8, or 10 days). The DBD treatments were applied using a DBD plasma system comprising a BK-130, step-up transformer (Phenix Technologies, Accident, MD, USA), two aluminum cyclic annular electrodes (150 mm diameter), and two dielectric barriers layers (polypropylene sheets). The trays were placed in the discharge area between two dielectric barriers and, when appropriate, treated for 60 s. The trays were stored at 4 °C for the duration of their storage period.

### 2.2. Microbiological Analysis

As separate batches, 4 sample cores per tray were minced together under aseptic conditions. From this preparation, 25 g of minced meat was weighed and aseptically transferred into a sterile stomacher bag with 225 mL of sterile 0.1% peptone PBS solution, and then blended with a BagMixer^®^ 400 stomacher (Interscience Ind., Puycapel, France) for 2 min. The resulting suspension was serially diluted with 0.1% peptone PBS (Phosphate-Buffered Saline) solution. To enumerate the non-injured cells, 100 μL aliquots were spread on the following agars: (1) Tryptic Soy Agar (TSA, Hope Bio-Technology Co., Ltd., Qingdao, China) incubated at 37 °C for 48 h, for the enumeration of viable aerobic flora; (2) *Pseudomonas* Cetrimide-Fusidin-Cephaloridine Selective Agar (CFC, Hope Bio-Technology Co. Ltd., Qingdao, China) incubated at 25 °C for 48 h, for the enumeration of *Pseudomonas* spp.; (3) Violet Red Bile Glucose Agar (VRBGA, Hope Bio-Technology Co., Ltd., Qingdao, China) incubated at 37 °C for 48 h, for the enumeration of *Enterobacteriaceae*; (4) De Man-Rogosa-Sharpe Agar (MRS, Hope Bio-Technology Co., Ltd., Qingdao, China) incubated at 30 °C for 48 h, for the enumeration of LAB; and (5) Streptomycin Sulphate Thallous Acetate cycloheximide (actidione) Agar (STAA, Hope Bio-Technology Co., Ltd., Qingdao, China) incubated at 25 °C for 48 h, for the enumeration of *B. thermosphacta*.

To enumerate the total cells, i.e., both injured and non-injured cells, on pork, the TAL method was employed [26]. The TAL method involves usinh a selective medium (SM) overlaid by a non-selective medium. Here, 14 mL (7 mL/7 mL; two times overlay) of melted tryptic Soy Agar was overlaid onto 25 mL of a pre-poured and solidified specific selective medium. The injured cells can resuscitate and grow on top layer (TSA) within the first few hours of incubation; then, the selective agents from the bottom layer (specific selective medium) can diffuse to the TSA layer and the resuscitated target cells can react with the selective agents to develop typical reactions, while other cells are inhibited by the selective agents [27,28]. Aliquots of 1000 or 100 μL were directly spread onto the TAL medium and incubated at corresponding conditions before counting the colonies. The sublethal ratios of bacteria after being exposed to DBD plasma were calculated as per Equation (1):

(1) $$\mathrm{Sublethal}\;\mathrm{ratio}\;(\%)=\frac{counts\;on\;TAL\;medium-counts\;on\;selective\;medium}{counts\;on\;TAL\;medium}$$

### 2.3. Modeling Growth Kinetics

To model growth kinetics of bacteria growth on pork during storage, the Baranyi and Roberts model [29] was applied (Equation (2)):

(2) $$y_{t}=y_{0}+\mu_{max}\times A(t)-ln(1+\frac{e^{\mu_{max}\times A\left(t\right)}-1}{e^{y_{max}-y_{0})}})$$

where y_t_ is the cell concentration in log_10_ CFU/g at time t, y_0_ represents the initial cell concentration; y_max_ is the maximum cell concentration; μ_max_ is the maximum specific growth rate in log_10_ CFU/d; A(t) is the lag phase described by Equation (3):

(3) $$A\left(t\right)=t+\frac{1}{v}\times ln(e^{-v\times t}+e^{-h_{0}}-e^{\left(-v\times t-h_{0}\right)})$$

where vis the rate of increase in the limiting substrate, assumed to be equal to μ_max_; h_0_ is the product of μ_max_ and λ; λ is the lag phase duration and represents the cells’ adjustment to the new environment [30]. The goodness of fit for the data was evaluated via the coefficient of determination (R^2^) and standard error of fit (*SE of Fit*), which was provided by DMFit.

The generation time was calculated in accordance with Reid et al. [31], and the calculation is described in Equation (4):

(4) $$G=\frac{t}{3.3\times {log}_{10}^{\frac{b}{B}}}$$

where G = generation time, t = time interval in days, b = number of bacteria at the end of t, and B = number of bacteria at the beginning of t.

### 2.4. Modified Lotka–Volterra Model

The modified Lotka–Volterra competition model is valuable in predictive microbiology for analyzing mixed microbial populations in homogeneous food products [32]. This is a straightforward model which can be represented using the following equations, Equations (5) and (6):

(5) $$\frac{dN_{A}}{dt}=\mu_{maxA}\frac{Q_{A}}{1+Q_{A}}\times \frac{N_{A}}{N_{maxA}}\times (N_{maxA}-N_{A}-\alpha_{AB}N_{B})$$

(6) $$\frac{dN_{B}}{dt}=\mu_{maxB}\frac{Q_{B}}{1+Q_{B}}\times \frac{N_{B}}{N_{maxB}}\times (N_{maxB}-N_{B}-\alpha_{BA}N_{A})$$

where N represent the quantities of colonies, μ_max_ represents the maximum specific growth rate, Q represent the physiological state of the cell, N_max_ represents the maximum population density and α_AB_ is a coefficient of interaction measuring the effects of cell B on cell A. At an interval [t_i−1_, t_i_], the model could be described using the following equations, Equations (7) and (8) [33,34]:

(7) $$lnN_{Ai}-lnN_{Ai-1}=\mu_{maxA}\times \frac{Q_{A}}{1+Q_{A}}\times \left(t_{i}-t_{i-1}\right)-\frac{\mu_{maxA}}{N_{maxA}}\times \int_{i-1}^{i}N_{A}(t)dt-\frac{\mu_{maxA}\alpha_{AB}}{N_{maxA}}\int_{i-1}^{i}N_{B}(t)dt$$

(8) $$lnN_{Bi}-lnN_{Bi-1}=\mu_{maxB}\times \frac{Q_{B}}{1+Q_{B}}\times \left(t_{i}-t_{i-1}\right)-\frac{\mu_{maxB}}{N_{maxB}}\times \int_{i-1}^{i}N_{B}(t)dt-\frac{\mu_{maxB}\alpha_{BA}}{N_{maxB}}\int_{i-1}^{i}N_{A}(t)dt$$

where N represents the quantities of colonies, μ_max_ represents the maximum specific growth rate, Q represents the physiological state of the cell, N_max_ represents the maximum population density and α_AB_ is a coefficient of interaction measuring the effects of cell B on cell A.

### 2.5. Statistical Analysis

Data were analyzed in SPSS (Version 20.0, SPSS, New York, NY, USA) using multiple one-way analysis of variance (ANOVA) and general linear regression (GLM) models. DBD treatment, storage period, culture methods, and their interactions were fitted as fixed effects, and a replicate was fitted as a random effect. Duncan’s multiple-range tests were applied to identify means of significant difference. The level of significance was set at 5% (*p* < 0.05).

The performance of models was evaluated by using the coefficient of determination (R^2^) and standard error of fit (*SE of Fit*). Generally, the closer R^2^ (Equation (9)) gets to 1, the higher the fitting degree of the equation. The *SE of Fit* (Equation (10)) quantifies the accuracy or precision of how well a mathematical model fits the observed data. It provides information about how closely the predicted values from the model align with the actual measurements. Essentially, a smaller *SE of Fit* indicates a better fit of the model to the data.

(9) $$R^{2}=1-\frac{\sum_{i=1}^{n}{\left(y_{o}-y_{p}\right)}^{2}}{\sum_{i=1}^{n}{\left(y_{o}-y_{m}\right)}^{2}}$$

(10) $$SE\;of\;Fit=\sqrt{\frac{\sum_{i=1}^{n}{\left(y_{o}-y_{p}\right)}^{2}}{n-f}}$$

where n is the number of observed points, f is the number of parameters estimated in the model, y_o_ and y_p_ represent the observed value and predicted value, and y_m_ represents the mean values of all samples at each detection point.

## 3. Results and Discussion

### 3.1. Variations in the Total Viable Counts of DBD-Treated Pork During Refrigerated Storage

**Figure 2:**
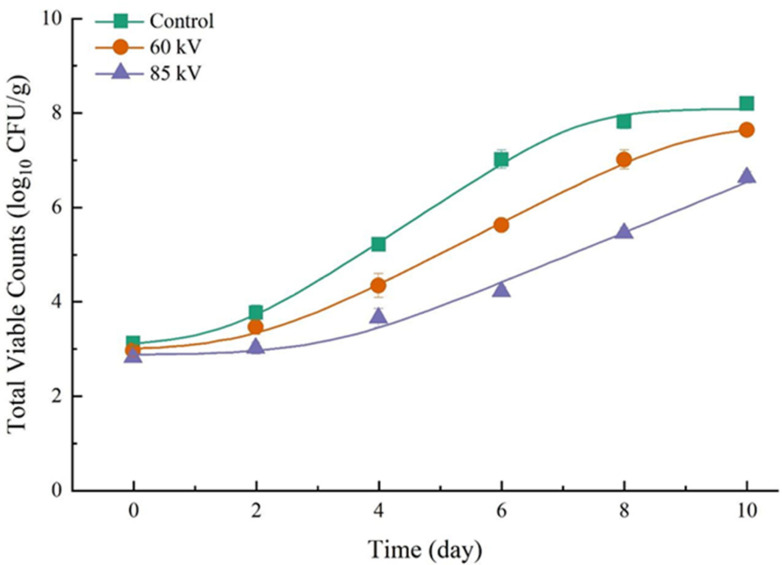
Total viable counts obtained for the pork loins exposed to different DBD treatments and held under refrigerated storage for up to 10 days (post-treatment). Plotted means (standard error shown as error bars) were calculated using the Baranyi and Roberts Model [41].

The objective of the microbiological analysis was to quantify the sublethal and lethal effect of DBD treatment on the indigenous microbiota population in the pork samples. The corresponding growth curves for TVC on pork during storage are presented in Figure 2. These show that, between Day 2 and Day 10 of the storage period, TVC was significant lower for the DBD-treated pork, and that an increase in DBD voltage led to more prominent inhibitory effects on the growth and reproduction of microorganisms. The TVC for the control, 60 kV, and 85 kV DBD-treated samples were 7.8, 7.0 and 5.4 log CFU/g, respectively, on Day 8 of the storage period. It is widely acknowledged that ROS and RNS, generated from cold plasma discharge, play a key role in the bactericidal effect of this non-thermal sterilization technology [35,36,37]. Under a higher voltage, the concentrations of ROS and RNS are also higher [38], meaning their bactericidal efficacy is increased. This outcome is the result of reactive species altering the permeability of the microorganism’s cell membranes and causing the oxidation of intracellular biomacromolecules [10,39]. However, differences between the TVC of control and DBD-treated samples were not observed at Day 0, confirming the results of Huang et al. [22] and Wang, Zhuang, Lawrence and Zhang [40].

The rough surface of meat can provide protective sites for bacteria to evade bactericidal treatments [42]. Microorganisms could migrate from the surface to depths up to 140 μm, through feather follicles, capillaries, and the routes formatted by the radial shrinkage of muscle fiber, where they are largely unaffected by bactericidal treatments [43,44]. From the Day 0 TVC results, it seems that DBD treatment does not offer any immediate bactericidal effect (*p* > 0.05). The growth rate of native bacteria on pork was, however, significantly inhibited post-treatment. This is evidenced in Table 1, which includes the pork TVC growth kinetic parameters (initial values, lag time, μ_max_). The R^2^ and *SE of Fit* were shown to represent the degree of kinetics compared to the reality. Samples exposed to 85 kV DBD treatments had lag times almost double those of control samples; the former’s μ_max_ was also significantly decreased when compared with that of the control sample (*p* < 0.05). In view of these results, sublethal damage to microorganisms, caused by DBD treatments, and the subsequent repair mechanism are likely to be responsible for the change in the dynamic of growth of TVC [37].

### 3.2. Pseudomonas spp., Enterobacteriaceae, LAB, and B. thermosphacta Counts in DBD-Treated Pork During Refrigerated Storage

*Pseudomonas* spp. and *B. thermosphacta* grow readily under packaging conditions with relatively high oxygen concentrations and are the main spoilage organisms in meat products [45]. In addition, bacteria of the *Enterobacteriaceae* family, especially some cryophiles, may become dominant spoilage organisms during the cooling and storage of raw meat products. It has been shown that spoilage bacteria of the genus LAB are mainly found in fresh meat products packaged in vacuum or air-conditioned packaging [46]. *Lactobacillus sakeus*, *Lactobacillus flexneri*, *Lactobacillus marinus*, *Lactobacillus reuteri*, and *Lactobacillus oligomerus* have been shown to be associated with the spoilage of certain fresh meats, and that these *Lactic acid bacteria* can cause severe acidification of meats, which can result in a rancid, sour taste and spoilage of the meat products [47]. Therefore, these four genera were chosen as the primary targets for microbial enumeration in this study during the refrigerated storage of pork.

The precise composition of indigenous microorganisms on meat and meat products is influenced by the type of meat, processing method, and storage conditions. This point withstanding, *Pseudomonas* spp., *Enterobacteriaceae*, *B. thermosphacta*, LAB were found to be the dominant microorganisms in the pork samples—aligning with previous research of meat held under low temperatures and in modified-atmosphere packaging [48,49,50,51].

**Figure 3:**
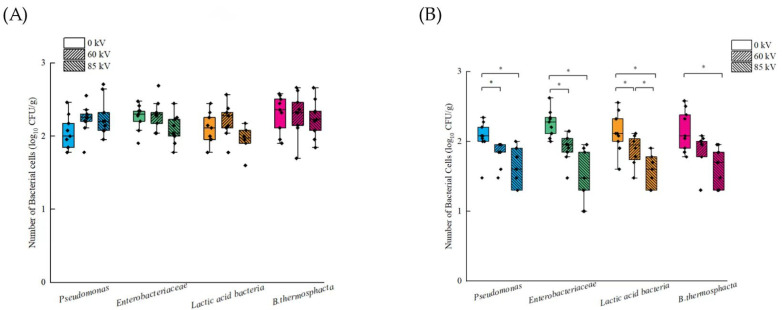
The *Pseudomonas* spp., *Enterobacteriaceae*, *Lactic acid bacteria* and *B. thermosphacta* counts on pork exposed to different levels of DBD. (**A**) The bacteria counts obtained by TAL method, and (**B**) the bacteria counts obtained on SM. * denotes significant differences (*p* < 0.05) between different treatment groups of the same strain.

For a more comprehensive insight into microorganisms’ response to the DBD treatment of pork, TAL and SM methods were applied to quantify the extent of sublethal injury of *Pseudomonas* spp., *Enterobacteriaceae*, LAB and *B. thermosphacta*. Figure 3A shows that DBD treatment had no significant bactericidal effect on *Pseudomonas* spp., *Enterobacteriaceae*, LAB, and *B. thermosphacta* when measured using the TAL method. Interestingly, when the SM method was employed, the 85 kV DBD treatment significantly decreased the population of *Pseudomonas* spp., *Enterobacteriaceae*, LAB and *B. thermosphacta* on pork by 0.4, 0.8, 0.5, and 0.5 log CFU/g, respectively. These results indicate a significant bactericidal effect of 85 kV DBD on pork for these four microorganisms (Figure 3B). Meanwhile, counts on DBD treated pork that were obtained using the SM method, irrespective of voltage and microorganism, were significantly lower than was observed using the TAL method. This finding may partially elucidate the seemingly contradictory research outcomes observed in previous studies, where DBD treatment did not demonstrate a significant bactericidal effect on indigenous microorganisms in meat but did demonstrate a sterilization effect on inoculated microorganism strains [44,52]. Within this context, the choice of cultivation method might play a substantive role.

Perni, Liu, Shama and Kong [53] found that cold plasma did not have a bactericidal effect when *E. coli* were recovered on TSA medium. However, when recovered on eosin methylene blue (EMB) medium, it was found that cold plasma could reduce *E. coli* by 1.0 log CFU/g, whereas on a more stringent medium, a reduction of 1.5 log CFU/g was achieved. Thus, it is inferred that cold plasma treatment can induce sublethal injury to microorganisms [53]. Smet et al. [54] systematically studied the effect of cold plasma on sublethal damage to *Salmonella* under different intrinsic factors (pH and salt concentration) and support system (liquid carrier and solid surface). Their results demonstrate the minor influence of the support system on *Salmonella* sublethal injury caused by cold plasma and the significant effects of the intrinsic factors to sublethal injury. Specifically, *Salmonella* cultured under incubation conditions of pH 5.5 and 6% NaCl suffered sensitive sublethal injury from cold plasma treatment than *Salmonella* cultured under incubation conditions of pH 7.0 and 0% NaCl [54]. It is apparent, however, that much focus has been on the sublethal injury of inoculated microorganisms, with few studies available on the sublethal injury of indigenous microorganism on food products caused by cold plasma treatment.

**Figure 4:**
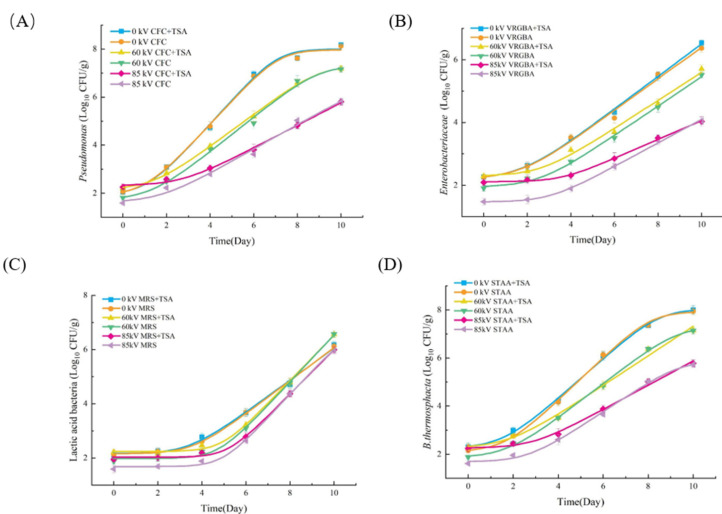
The recovery and growth of (**A**) *Pseudomonas*, (**B**) *Enterobacteriaceae*, (**C**) *Lactic acid bacteria*, and (**D**) *B. thermosphacta* on pork with and without DBD treatment during refrigerated storage for up to 10 days (post-treatment). All points are actual values, and all lines are fitted values of the model. Error bars represent the standard error.

The recovery and growth of *Pseudomonas*, *Enterobacteriaceae*, LAB, *B. thermosphacta* in pork exposed to different DBD treatments and stored for up to 10 days, post-treatment, determined by the SM and TAL method, are shown in Figure 4. The growth curves for these four microorganisms show a similar trend to TVC, whereby their growth and reproduction are inhibited by DBD, and this effect is more prominent under the higher-voltage treatment. It was also found that the difference in microorganism population, determined by the SM and TAL methods, decreased as the storage period increased, before somewhat equalizing in count by Day 10 of the storage period. This result suggests that DBD treatment induced the broad-spectrum sublethal injury of microorganisms on pork and that the injured microorganisms regained viability during the storage period. This shows a real risk of underestimating the presence of foodborne microorganisms, following DBD treatment, when using the SM method.

**Figure 5:**
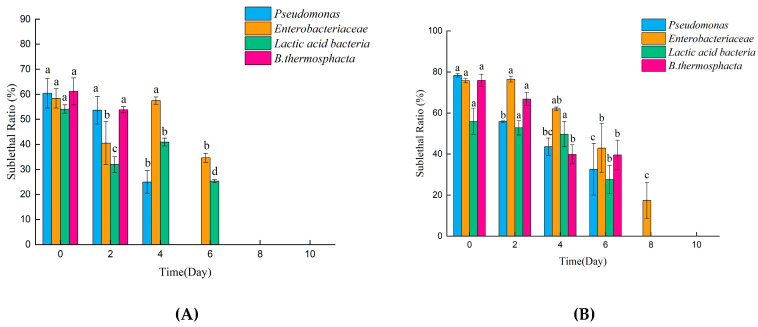
The changes in sublethal rates of *Pseudomonas* spp., *Enterobacteriaceae*, *Lactic acid bacteria* and *B. thermosphacta* on (**A**) 60 kV-DBD- and (**B**) 85 kV-DBD-treated pork held under refrigerated storage for up to 10 days (post-treatment). Error bars represent the standard error. Within the same bacterial genus, different lowercase letters at different times indicate significant differences (*p* < 0.05).

To confirm the effects of DBD treatment on the sublethal injury of *Pseudomonas* spp., *Enterobacteriaceae*, LAB and *B. thermosphacta* on pork, the variations in the sublethal injury rates of these four microorganisms were monitored across the 10-day storage period (Figure 5). For the 60 kV DBD treatment, the sublethal injury rate of *Pseudomonas* spp. and *B. thermosphacta* decreased with an increase in storage period. The injured *B. thermosphacta* persisted for at least 2 days into the total storage period, the injured *Pseudomonas* spp. persisted for at least 4 days, while the injured *Enterobacteriaceae* and LAB persisted for at least 6 days. At the 85 kV DBD treatment, the sublethal injury durations of these four microorganisms were longer than observed for the 60 kV DBD treatment. By Day 6, the sublethal injury rate decreased for *Pseudomonas* spp. (78.4 to 32.6%), *Enterobacteriaceae* (75.7 to 42.9%), LAB (55.9 to 27.7%), and *B. thermosphacta* (75.9 to 39.5%). Regardless of whether the DBD treatment was conducted at 60 kV or 85 kV, the sublethal rates of the four microorganisms decreased with increases in the storage period. This finding indicates that microorganism with sublethal injury have the capacity for self-renewal.

Following DBD treatment, microorganisms will be either alive, dead, or in a sublethal injured state. Plasma treatment causes injury to the structure or function of bacterial cell membranes, or a combination of both, including the destruction of membrane integrity and increased membrane permeability [55,56]. When a membrane is perforated but its metabolic activity is still active, a sublethal condition is present. In this case, the injured subpopulation can subsequently develop into a dead or alive microorganism, and the key to how they develop depends on the external environment. When external stressors are removed and microorganisms are in a nutrient-rich and favorable environment, some of the genes related to SOS response, nitrosative stress, the cell envelop-related response, and metabolism are found to be up-regulated, which contributes to the repair of damaged membranes [57,58]. Meanwhile, OxyR is an important regulatory protein. This protein is activated by ROS and upregulates the expression of antioxidant genes to prevent further damage by ROS to cells [55]. Further research has found that the during the SOS response, YneA accumulates within the cell, which inhibits septum formation and prevents cellular division [59,60,61]. This may be one of the reasons why sublethally injured microorganisms have an elongated lag phase. Sublethally injured microorganisms require energy to repair the damaged membrane, maintain osmotic pressure, and express selective genes [62]. Therefore, sufficient and complex nutrients are required for their recovery [63]. In addition to nutrients, certain minerals have been proposed to be beneficial for promoting the recovery of injured microorganisms. For example, Mn cations participate in the adjustment of the proteome, and the adjusted proteins participate in DNA repair, oxidoreductase activity, and the remodeling of gene expression [64]. Zn cations can enhance the activity of Cu/Zn superoxide dismutase, alleviate oxidative stress, and likewise promote microorganism recovery [65].

The lag phase is an adjustment period during which the microorganisms undergo some regulation to survive and thrive better in the new environment [66]. This parameter indicates the physiological state of the microorganisms, and thus can provide a better insight into their basic state [67]. DBD treatment caused an extension to the lag phase of *Pseudomonas* spp., *Enterobacteriaceae*, LAB and *B. thermosphacta*. Furthermore, the higher the treatment voltage, the longer the lag phase observed. These results suggest that DBD treatment prolonged the time required for these four microorganisms to adapt to the new environment. For the DBD-treated samples, the lag phase of the microorganisms cultured by the TAL method was longer than that of the microorganisms cultured by the SM method, and this phenomenon was more pronounced under higher-voltage treatment. During the 85 kV DBD treatment, the lag phases of *Pseudomonas* spp., *Enterobacteriaceae*, LAB and *B. thermosphacta* cultured by the TAL method were 2.5, 3.7, 4.3, and 3.0 days, respectively, while the lag phases of *Pseudomonas* spp., *Enterobacteriaceae*, LAB and *B. thermosphacta* cultured by the SM method were 1.6, 3.0, 3.6, and 2.4 days, respectively.

The maximum specific growth rate is a critical growth kinetic parameter and used to describe the growth behavior of microorganisms on food [68]. The maximum specific growth rate can vary depending on the type of microorganism, the environmental conditions, and the limiting substrate [49,69]. Different microorganisms have different maximum specific growth rates, which reflect their physiological and metabolic capabilities [70]. Table 2 shows that DBD treatment significantly affects the maximum specific growth rate of *Pseudomonas* spp., *Enterobacteriaceae* and *B. thermosphacta*; and this effect becomes more obvious with increasing treatment voltage. Using the TAL method, the maximum specific growth rates of *Pseudomonas* spp., *Enterobacteriaceae* and *B. thermosphacta* in the control group were found to be 1.0, 0.5, and 0.9, respectively. After 60 kV DBD treatment, the maximum specific growth rates of *Pseudomonas* spp., *Enterobacteriaceae* and *B. thermosphacta* in pork were shown to significantly decrease to 0.7, 0.5, and 0.7, respectively. As the DBD voltage increased to 85 kV, the maximum specific growth rates of *Pseudomonas* spp., *Enterobacteriaceae* and *B. thermosphacta* decreased significantly to 0.4, 0.38, and 0.6, respectively. The results obtained using the TAL method and the SM method did not significantly differ, except for the *Enterobacteriaceae* exposed to 85 kV DBD. However, DBD treatment had no significant effect on the maximum specific growth rate of LAB (*p* > 0.05).

The lag phase and the maximum specific growth rate contribute to our understanding of the physiological state of microorganisms. Pina-Perez, Martinet, Palacios-Gorba, Ellert and Beyrer [37] evaluated the influence of plasma treatment on the dynamics of growth of *Bacillus subtilis* using the Gompertz model. Their results indicated significant differences in the lag phase and maximum specific growth rate between the treatment and control groups, with the lag phase differences becoming more evident as the plasma power density increased [37]. Han, Boehm, Patil, Cullen and Bourke [57] revealed that the maximum specific growth rate of plasma-treated *E. coli*, *L. monocytogenes*, and *S. aureus* decreased as plasma treatment times increased [71]. In general, plasma treatment results in lipid and protein oxidation, with implications on the changes in cell membrane fluidity and enzyme activity [72]. These alterations are considered to impact the lag phase and the maximum specific growth rate of microorganisms [73,74]. Table 2 shows that the maximum specific growth rate of LAB was not affected by DBD treatment, unlike that of *Pseudomonas* spp., *Enterobacteriaceae* and *B. thermosphacta*. DBD treatment did, however, significantly reduced the generation time of LAB on pork. This raised the following question: does DBD treatment improve the ability of LAB to adapt to colonies and enhance its competitiveness?

### 3.3. Interactions Between Pseudomonas spp., Enterobacteriaceae, LAB and B. thermosphacta in DBD-Treated Pork

**Figure 6:**
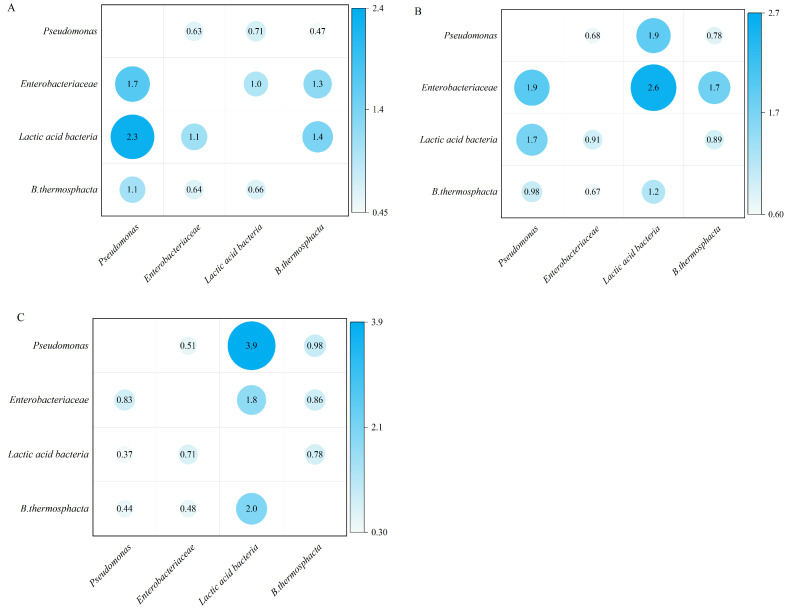
Interaction of *Pseudomonas* spp., *Enterobacteriaceae*, *Lactic acid bacteria* and *B. thermosphacta* on (**A**) control, (**B**) 60 kV DBD-treated pork, and (**C**) 85 kV DBD pork based on the Lotka–Volterra model.

Modified Lotka–Volterra Models were used to evaluate the dynamic interactions between the *Pseudomonas* spp., *Enterobacteriaceae*, LAB, and *B. thermosphacta* (Figure 6). For control samples, the coefficients of interaction of LAB on *Pseudomonas* spp., *Enterobacteriaceae*, and *B. thermosphacta* were α_12_ = 0.7, 1.0, and 0.7, respectively, and α_21_ = 2.3, 1.1, and 1.4, respectively. As α_12_ < α_21_, it was concluded that the influence of LAB on *Pseudomonas* spp., *Enterobacteriaceae*, and *B. thermosphacta* was much lower than that of *Pseudomonas* spp., *Enterobacteriaceae*, and *B. thermosphacta* on LAB. For pork exposed to 60 kV DBD, the coefficient of interaction of LAB on *Pseudomonas* spp., *Enterobacteriaceae*, and *B. thermosphacta*, α_12_ decreased to 1.9, 2.6 and 1.2, respectively, and α_21_ increased to 1.7, 0.9 and 0.9, respectively. These indicate the influence of LAB on *Pseudomonas* spp.; *Enterobacteriaceae*, and *B. thermosphacta* was enhanced, while the influence of *Pseudomonas* spp., *Enterobacteriaceae*, and *B. thermosphacta* on LAB was lessened. These results present that DBD enhances the antagonistic activity of LAB during pork storage—an outcome confirmed by Zhao et al. [75], who found the proportion of *Pseudomonas* in plasma-treated chicken meat increased during storage, while the proportion of *Carnobacterium* decreased. ROS generated by plasma can oxidize the lipids in the outer membrane, leading to the inactivation of Gram-negative bacteria and an increase in the proportion of Gram-positive bacteria, and the higher the initial load of microorganisms, the higher the proportion in the latter storage periods [75]. Previous studies have shown that plasma treatment can cause a decrease in surface pH [15,76,77], which may create an environment that is more suited for LAB compared to other microorganisms. In addition, when grown in associated cultures, LAB exerted an antagonistic action on the growth of *Staphylococcus aureus*, *Salmonella typhimurium* and *E. coli* [67]. The metabolic product of LAB, lactic acid, also has a bacteriostatic effect, and the bacteriostatic effect of lactic acid on plasma-treated microorganisms is enhanced [20,78]. However, it should be noted that CFC, STAA, and VRBGA media all contain one or more bactericidal agents (i.e., cetrimide, streptomycin sulfate, bile salts), while MRS media primarily select LAB via pH adjustments, which may lead to a higher detection rate of LAB.

## 4. Conclusions

This study proves that DBD treatment will cause damage to the indigenous microorganisms on pork and will extend its shelf-life when refrigerated and in modified-atmosphere packaging. However, when using conventional detection methods, this sublethal damage can lead to type II error. Adopting modified culture methods can help avoid this problem, to some extent, and make the detection results more accurate. DBD treatment also causes changes in the succession of the microorganisms in the pork during storage, decreasing the proportion of *Pseudomonas* spp. and *B. thermosphacta*, and increasing the proportion of LAB. This bactericidal characteristic as well as its effects on the preservation and increase in the proportion of LAB suggests that DBD treatment can likewise prolong the preservation of fermented meat products. Additional research is, nonetheless, advisable given the current experimental conditions and the extrinsic and intrinsic factors that impact microorganism growth and population.

## Figures and Tables

**Table 1:** Lag time (λ) and maximum specific growth rate (μ_max_) for total viable counts according to the Baranyi and Roberts model [41].

DBD ^A^ (kV)	Initial Value (log_10_CFU/g)	λ ^B^ (d)	μ_max_ ^C^ (d^−1^)	R^2 D^	*SE of Fit* ^E^
0	3.12 ± 0.09 ^a^	1.52 ± 0.08 ^b^	0.86 ± 0.02 ^a^	0.995	0.151
60	2.95 ± 0.12 ^a^	1.82 ± 0.14 ^b^	0.64 ± 0.03 ^b^	0.996	0.116
85	2.81 ± 0.11 ^a^	3.12 ± 0.13 ^a^	0.53 ± 0.01 ^c^	0.986	0.177

^A^ DBD: dielectric barrier discharge plasma treatment time; ^B^ λ: the lag phase duration; ^C^ μ_max_: the maximum specific growth rate. ^D^ R^2^: the coefficient of determination; ^E^ *SE of Fit:* standard error of fit. Different lower case letters, ^a,b,c^, denote significant differences (*p* < 0.05) between different treatment groups. Results are expressed as the means ± standard error.

**Table 2:** Lag time (λ), maximum specific growth rate (μ_max_), and generation time for *Pseudomonas*, *Enterobacteriaceae*, LAB, and *B. thermosphacta* according to the Baranyi and Roberts model [41].

DBD ^A^ (kV)		Medium	λ ^B^ (d)	μ_max_ ^C^ (d^−1^)	Generation Time (d)	R^2 D^	*SE of Fit* ^E^
*Pseudomonas*	0	TAL	1.14 ± 0.13 ^b^	1 ± 0.02 ^a^	0.48 ± 0.01 ^c^	0.988	0.274
		SM	1.19 ± 0.05 ^b^	0.95 ± 0.01 ^a^	0.51 ^bc^	0.99	0.229
	60	TAL	1.35 ± 0.12 ^b^	0.67 ± 0.01 ^b^	0.57 ± 0.01 ^b^	0.997	0.111
		SM	1.28 ± 0.03 ^b^	0.72 ± 0.03 ^b^	0.55 ± 0.01 ^b^	0.983	0.275
	85	TAL	2.47 ± 0.18 ^a^	0.44 ± 0 ^c^	0.77 ± 0.02 ^a^	0.994	0.099
		SM	1.61 ± 0.32 ^b^	0.5 ± 0.03 ^c^	0.73 ± 0.03 ^a^	0.971	0.263
*Enterobacteriaceae*	0	TAL	2.31 ± 0.06 ^cd^	0.51 ± 0.01 ^a^	0.63 ± 0.01 ^c^	0.992	0.142
		SM	1.96 ± 0.13 ^d^	0.5 ± 0.01 ^a^	0.66 ± 0.01 ^c^	0.985	0.192
	60	TAL	3.54 ± 0.06 ^a^	0.46 ± 0.01 ^b^	0.81 ± 0.13 ^bc^	0.987	0.14
		SM	2.56 ± 0.18 ^bc^	0.47 ± 0.01 ^b^	0.68 ± 0.01 ^c^	0.996	0.093
	85	TAL	3.72 ± 0.05 ^a^	0.28 ± 0.01 ^d^	1.21 ± 0.03 ^a^	0.978	0.104
		SM	2.96 ± 0.14 ^b^	0.36 ± 0.01 ^c^	0.94 ± 0.01 ^b^	0.99	0.102
LAB ^F^	0	TAL	2.34 ± 0.25 ^d^	0.77 ± 0.07 ^a^	0.53 ± 0.01 ^a^	0.991	0.213
		SM	2.54 ± 0.08 ^d^	0.74 ± 0.03 ^a^	0.53 ± 0.01 ^a^	0.987	0.244
	60	TAL	3.56 ± 0.03 ^b^	0.83 ± 0.01 ^a^	0.49 ± 0.01 ^b^	0.994	0.164
		SM	3.03 ± 0.05 ^c^	0.81 ± 0.02 ^a^	0.54 ± 0.01 ^a^	0.985	0.28
	85	TAL	4.31 ± 0.14 ^a^	0.79 ± 0.04 ^a^	0.46 ± 0.01 ^c^	0.99	0.178
		SM	3.55 ± 0.18 ^b^	0.74 ± 0.01 ^a^	0.47 ± 0.01 ^bc^	0.991	0.184
*B. thermosphacta*	0	TAL	1.75 ± 0.06 ^c^	0.87 ± 0.01 ^a^	0.47 ± 0.01 ^b^	0.997	0.126
		SM	1.79 ± 0.12 ^c^	0.93 ± 0.02 ^a^	0.46 ^b^	0.998	0.101
	60	TAL	2.46 ± 0.16 ^b^	0.73 ± 0.03 ^b^	0.49 ± 0.01 ^b^	0.988	0.225
		SM	1.8 ± 0.05 ^c^	0.72 ± 0.01 ^b^	0.47 ± 0.01 ^b^	0.999	0.083
	85	TAL	3.01 ± 0.17 ^a^	0.59 ± 0.04 ^c^	0.64 ± 0.01 ^a^	0.997	0.081
		SM	2.36 ± 0.33 ^b^	0.58 ± 0.08 ^c^	0.61 ± 0.02 ^a^	0.976	0.272

^A^ DBD: dielectric barrier discharge plasma treatment time; ^B^ λ: the lag phase duration; ^C^ μ_max_: the maximum specific growth rate; ^D^ R^2^: the coefficient of determination; ^E^ *SE of Fit:* standard error of fit; ^F^ LAB: lactic acid bacteria. Different lower case letters, ^a,b,c,d^, denote significant differences (*p* < 0.05) between different treatment groups of the same strain. Results are expressed as the means ± standard error.

## Data Availability

Data will be made available on request.
